# The Importance of Cellular Therapy in the Clinical Case of a Young Man With a Challenging Precursor B-cell Lymphoblastic Leukemia

**DOI:** 10.7759/cureus.75730

**Published:** 2024-12-15

**Authors:** Catarina F Almeida, Liliana Fonseca, Inês Ramos, Fátima Amado, Lúcia Vieira, Sara B Lopes, Ana Salselas, Susana Roncon

**Affiliations:** 1 Immunohemotherapy Department, Unidade de Saúde Local da Região de Aveiro, Aveiro, PRT; 2 Immunohemotherapy Department, Unidade Local de Saúde de Viseu Dão-Lafões, Viseu, PRT; 3 Hematology Department, Instituto Português de Oncologia do Porto Fernando Gentil, Entidades Públicas Empresariais (EPE), Porto, PRT; 4 Cellular Therapy Department, Instituto Português de Oncologia do Porto Fernando Gentil, Entidades Públicas Empresariais (EPE), Porto, PRT; 5 Immunohemotherapy Department, Instituto Português de Oncologia do Porto Fernando Gentil, Entidades Públicas Empresariais (EPE), Vila Nova de Gaia, PRT

**Keywords:** cellular therapy, hematologic illnesses, innovative approaches, processing and cryopreservation, product collection, treatment

## Abstract

Acute lymphoblastic leukemia (ALL) poses a significant challenge due to its high relapse rate despite initial chemotherapy. Cell therapy plays an important and promising role in refractory ALL cases.

The aim is to present a complex case of a 20-year-old male patient with relapsed ALL and to explore the different therapeutic options of cellular therapy - allogeneic hematopoietic stem cell transplantation, donor lymphocyte infusion, and chimeric antigen receptor T - detailing the collection, processing, and infusion, as well as the associated complications and management strategies. Data were collected from patient records.

This case highlights the evolving landscape of immunotherapy in hematological malignancies and emphasizes the importance of optimizing cellular therapy approaches for improved patient outcomes.

## Introduction

Acute lymphoblastic leukemia (ALL) is a clonal hematologic disorder characterized by excessive proliferation and impaired differentiation of leukemic blasts. The primary treatment for ALL in adults typically involves long-term chemotherapy. Although 80-90% of adults achieve complete or partial remission, the risk of relapse or refractory leukemia remains high (~50%), resulting in an overall cure rate of approximately 40% [1,2].

The purpose of this letter is to present the challenging case of a young patient with relapsed ALL and to highlight the different therapeutic options that cellular therapy can offer in the management of these cases.

## Case presentation

A 20-year-old male patient weighing 58 kg was diagnosed with precursor B-cell acute lymphoblastic leukemia (B-ALL) with amplification of the RUNX1 gene in April 2016. Four months after an intensive chemotherapy regimen - Berlin-Frankfurt-Munster (BFM-90) protocol - relapse occurred, and the patient was proposed for second-line chemotherapy protocol - ALL-REZ-BFM - followed by allogeneic bone marrow transplantation with a myeloablative conditioning regimen.

The patient has a younger brother who is a 10/10 human leukocyte antigen (HLA) match and was proposed for peripheral blood stem cell (PBSC) collection in January 2021.

The donor was 17 years old, weighed 60 kg, and had an isogroup blood type. He presented no significant past medical history and no regular medications. Serologies for human immunodeficiency virus (HIV), hepatitis B virus (HBV), and hepatitis C virus (HCV), were nonreactive, and nucleic acid tests (NAT) were negative. Epstein Barr (EBV), cytomegalovirus (CMV), human T-lymphotropic virus (HTLV), herpes simplex virus type 1 and type 2, syphilis, and toxoplasmosis were also nonreactive. Apheresis was planned to harvest ≥ 4x106 CD34+ cells/kg in a total of ≥ 232x106 CD34+ cells. Mobilization was performed for five days with 480 micrograms of granulocyte-colony stimulating factor (G-CSF) every 12 hours (16mcg/kg/day) without complications. On the day of apheresis, he presented a hemoglobin of 15.1g/dL, white blood cells were 62.03x109/L, platelets were 200x109/L, and CD34+ cell count was 124/µL. With a calculated total blood volume of 4370 mL, 3.5 blood volumes were processed via double peripheral venous access, and the extracorporeal circuit was anticoagulated with heparin and citrate (proportion of blood to anticoagulant of 25:1). The procedure took 244 minutes without complications. The hematopoietic graft has a volume of 225 mL, 1230.3x108 CD45+ cells, 127.2x108 T lymphocytes, and 999.5x106 CD34+ cells. A split was performed; 85mL were infused with >4x106 CD34+ cells per kg of the recipient as requested by the attending physician, and the remainder was divided into four bags and cryopreserved at -196ºC in liquid nitrogen. Graft quality control was ensured with cell content >4x106/kg CD34+ cells, granulocyte-macrophage colony-forming unit (CFU-GM) >1x105/kg (1427.2 x105), and negative microbiology.

In the pre-infusion period, the patient underwent a conditioning regimen with busulfan and cyclophosphamide and graft-versus-host disease (GVHD) prophylaxis with cyclosporine A and methotrexate. On the same day as the sibling apheresis, the patient received 4.72x106 CD34+cells/Kg and 0.82x108 CD3+cells/Kg with no reported adverse events. Until discharge, there was an episode of febrile neutropenia without focus or isolated agent and grade I oropharyngeal and esophageal mucositis, which resolved after therapy. The patient remained hospitalized for 27 days and demonstrated neutrophil engraftment (>0.5x109/L) on post-transplant day +15. Bone marrow evaluations at one and three months post-allo-hematopoietic stem cell transplantation showed complete chimerism and negative measurable residual disease by immunophenotyping and no infectious complications. After five months, in May 2021, the patient developed grade I cutaneous GVHD (malar erythema) that responded to corticosteroid therapy. At the six-month evaluation, despite maintaining complete chimerism in the bone marrow, medullary hypocellularity was observed with 0.23% blast cells. Donor lymphocyte infusion (DLI) was proposed as a preemptive treatment strategy for ALL relapse.

The patient received four DLIs (each with >1x107 CD3+ cells/kg) at four different time points over a period of six months. Evaluation after the fourth infusion revealed criteria for ALL relapse with 38.5% blasts on myelogram and 6.1% on immunofluorescence. Complete chimerism was maintained for polymorphonuclear (PMN) cells and 87% for mononuclear (MN) cells. Cytogenetics showed that 7% of the 200 interphase nuclei analyzed had three to four copies of the RUNX1 gene (21q22). There was no evidence of GVHD.

The multidisciplinary team decided to propose to the patient chimeric antigen receptor T (CAR-T) cell therapy - tisagenlecleucel. Despite being 26 years old at the time and the upper age limit for eligibility being 25, the patient was approved for treatment. In March 2022, weighing 56 kg, six weeks after the last DLI infusion, autologous peripheral blood mononuclear cells were collected by apheresis with the aim of obtaining ≥2x109 total nucleated cell count, ≥1x109 CD3+ cells and 3% CD3+ cells.

Analytically, on the day of apheresis, hemoglobin was 15.1g/dL, white blood cells were 7.04x109/L, platelets were 146x109/L, and CD3+ cell count in peripheral blood was 734µL. Serologies for HIV, HCV, HBV, HTLV, Syphilis, EBV, and CMV were nonreactive, and NAT was undetectable. Slightly more than 10L were processed via peripheral venous access with anticoagulation of the extracorporeal circuit with citrate (ratio of blood to anticoagulant of 14:1). The procedure took 199 minutes, and the patient experienced facial paresthesia that improved with the administration of intravenous calcium gluconate.

Since the final product harvested had a total volume of 175mL, centrifugation techniques were used to obtain a volume reduction to 49mL. Afterward, it was added 62 mL of albumin and 12 mL of DMSO and the final volume (123ml) was divided into two bags, which were cryopreserved at -150ºC in the gaseous phase of nitrogen. Cellular control of the product was the following: 103.3 x 108 leukocytes, 49.6 x 108 T lymphocytes, with CD45+ viability of 99% and CD3+ viability of 100%. The microbiological culture was negative.

Two days after harvest, one of the bags was shipped to the manufacturing site according to the required safety and quality measures [3], while the other remained stored at the hospital.

Taking into account the pharmaceutical company's requirements, the mononuclear cells were approved for the manufacture of advanced therapy medicinal products, and 27 days after harvest, the CAR-T cells were shipped with a dose of 1.3x108 of transduced viable T-cells. Four days after reception, they were thawed and infused.

Prednisone, etoposide, procarbazine, and cyclophosphamide were administered as a bridge to CAR-T cell therapy with a good metabolic response. Lymphodepleting chemotherapy was performed with fludarabine (30mg/m2) for four days and cyclophosphamide (500mg/m2) for two days. CAR T-cell infusion was performed two days after completion of lymphodepletion, without any complications reported. Before the infusion, hemoglobin was 11.3g/dL, white blood cells were 0.44x109/L, and platelets were 87x109/L. After the infusion (day +1), the patient developed intermittent fever, but no severe cytokine release syndrome (CRS) was observed. Corticosteroids and empiric antibiotics were started with dexamethasone and piperacillin-tazobactam, respectively. On day +5, he developed a fever with hypotension (CRS grade 2), and the antibiotics were changed to meropenem and vancomycin. On day +9, due to persistent fever despite hemodynamic stability (CRS grade 1), a single dose of tocilizumab 4 mg/kg was administered. No immune effector cell-associated neurotoxicity syndrome (ICANS) was observed. On day +10, leukocytes increased to 1.93x109/L with neutrophils 0.23x109/L and lymphocytes 1.68x109/L with stable platelet and hemoglobin levels. He was discharged from the hospital on day +20 without infectious complications.

One year after the infusion, the patient has complete peripheral blood chimerism for PMN, MN, T lymphocytes, and B lymphocytes with a myelogram showing no excess blasts or dysplasia and negative measurable residual disease by immunophenotyping. To date, the patient remains in complete remission and had no infectious complications or evidence of GVHD, maintaining frequent follow-ups.

## Discussion

Cellular therapy has emerged as a pivotal approach for treating hematologic malignancies, particularly in cases of relapsed or refractory ALL. The case presented here exemplifies the complex therapeutic strategies needed for a 20-year-old male patient with relapsed ALL, focusing on the potential of cellular therapies such as allogeneic stem cell transplantation, DLI, CAR-T cell therapy [u1]. Allogeneic hematopoietic stem cell transplantation remains a cornerstone of treatment for ALL relapses, offering the possibility of immune-mediated eradication of leukemia cells due to the graft-versus-leukemia effect. However, the occurrence of GVHD and relapse after initial engraftment, as seen in this patient, remains a significant challenge in achieving sustained remission [4].

Recent advancements in CAR-T cell therapy, particularly with products like tisagenlecleucel, have shown promising results in treating patients with refractory B-cell malignancies, including B cell ALL. Clinical trials and studies have demonstrated that CAR-T cells can provide long-lasting remission and even cure in a subset of patients. A systematic review and meta-analysis demonstrated that CAR-T cell therapy offers a high overall response rate, with approximately 80% of patients achieving complete remission after treatment [5]. This has been confirmed in several other studies, reinforcing CAR-T's potential to transform the landscape of leukemia treatment.

The role of cellular therapies also extends to optimizing the collection, processing, and cryopreservation of cellular products to ensure maximal therapeutic benefit. The careful management of these processes, as evidenced by the patient's stem cell collection, CAR-T cell processing, and storage protocols, is essential in maintaining the quality of the product and the efficacy of the treatment.

Despite their transformative potential, cellular therapies are associated with significant financial and logistical challenges that can exacerbate disparities in access to treatment. The high costs of manufacturing, processing, and administering therapies such as CAR-T cells, coupled with the need for specialized facilities and highly trained personnel, can create substantial barriers. These factors contribute to inequities in availability and accessibility.

## Conclusions

As the field of immunotherapy continues to expand, there is increasing evidence supporting its role in improving survival rates and minimizing relapse in patients with hematologic malignancies. The results from this case underscore the importance of coordinated care, rigorous monitoring, and specialized techniques to improve patient outcomes and ensure the successful integration of cellular therapy into clinical practice.

Continued research and clinical trials are necessary to refine these approaches, reduce side effects, and enhance the accessibility and efficacy of cellular therapies. The integration of these therapies into routine clinical practice is likely to become a standard of care in the near future, offering hope to patients facing otherwise limited treatment options. As well, there is an urgent need for policy interventions, funding mechanisms, and global collaborations aimed at reducing costs and promoting equitable access to these life-saving treatments.
